# Evaluation of the MIM Symphony treatment planning system for low‐dose‐rate‐ prostate brachytherapy

**DOI:** 10.1120/jacmp.v16i5.5057

**Published:** 2015-09-08

**Authors:** Sandeep K. Dhanesar, Tze Y. Lim, Weiliang Du, Teresa L. Bruno, Steven J. Frank, Rajat J. Kudchadker

**Affiliations:** ^1^ Department of Radiation Physics The University of Texas MD Anderson Cancer Center Houston TX; ^2^ The University of Texas at Houston Graduate School of Biomedical Sciences Houston TX; ^3^ Department of Radiation Oncology The University of Texas MD Anderson Cancer Center Houston TX USA

**Keywords:** treatment planning, prostate brachytherapy, MIM Symphony, VariSeed

## Abstract

MIM Symphony is a recently introduced low‐dose‐rate prostate brachytherapy treatment planning system (TPS). We evaluated the dosimetric and planning accuracy of this new TPS compared to the universally used VariSeed TPS. For dosimetric evaluation of the MIM Symphony version 5.4 TPS, we compared dose calculations from the MIM Symphony TPS with the formalism recommended by the American Association of Physicists in Medicine Task Group 43 report (TG‐43) and those generated by the VariSeed version 8.0 TPS for iodine‐125 (I‐125; Models 6711 and IAI‐125A), palladium‐103 (Pd‐103; Model 200), and cesium‐131 (Cs‐131; Model Cs‐1). Validation was performed for both line source and point source approximations. As part of the treatment planning validation, first a QA phantom (CIRS Brachytherapy QA Phantom Model 045 SN#D7210‐3) containing three ellipsoid objects with certified volumes was scanned in order to check the volume accuracy of the contoured structures in MIM Symphony. Then the DICOM data containing 100 patient plans from the VariSeed TPS were imported into the MIM Symphony TPS. The 100 plans included 25 each of I‐125 pre‐implant plans, Pd‐103 pre‐implant plans, I‐125 Day 30 plans (i.e., from 1 month after implantation), and Pd‐103 Day 30 plans. The dosimetric parameters (including prostate volume, prostate D90 values, and rectum V100 values) of the 100 plans were calculated independently on the two TPSs. Other TPS tests that were done included verification of source input and geometrical accuracy, data transfer between different planning systems, text printout, 2D dose plots, DVH printout, and template grid accuracy. According to the line source formalism, the dosimetric results between the MIM Symphony TPS and TG‐43 were within 0.5% (0.02 Gy) for r>1 cm. In the line source approximation validation, MIM Symphony TPS values agreed with VariSeed TPS values to within 0.5% (0.09 Gy) for r>1 cm. Similarly, in point source approximation validation, the MIM Symphony values agreed to within 1% of the TG‐43 and VariSeed values for r>1 cm. The volume calculations obtained from the MIM Symphony TPS for the CIRS Brachytherapy QA Phantom were within 1% of the actual volume of the phantom. For the clinical cases, the volume and dosimetric parameter calculations for the prostate and rectum did not differ substantially between the pre‐implant and Day 30 plans. Overall, our investigations showed negligible differences in dosimetry calculations and planning parameters between the two TPSs. The tests done to check the performance of the MIM Symphony TPS, such as the library data, data transfer, isodose and DVH printout, were found to be satisfactory. On the basis of these results, we conclude that the MIM Symphony TPS can be used as an alternative to the VariSeed TPS for low‐dose‐rate prostate brachytherapy.

PACS numbers: 87.53.Jw; 87.55.D‐, 87.55.Qr

## I. INTRODUCTION

Prostate cancer is the most common malignancy in men in the United States. An estimated 238,590 new cases were diagnosed in 2013.^(1)^ The number of newly diagnosed cases continues to increase owing to early prostate cancer detection enabled by prostate‐specific antigen screening and changes in population demographics. Several treatment options are available depending on the extent of the disease. Low‐dose‐rate (LDR) prostate brachytherapy is of particular interest for the treatment of early‐stage prostate cancer. For more advanced disease, LDR prostate brachytherapy may be used in conjunction with external‐beam radiation therapy. LDR prostate brachytherapy involves permanent interstitial implantation of radioactive seeds, such as iodine‐125 (I‐125), palladium‐103 (Pd‐103), or cesium‐131 (Cs‐131), under imaging and template guidance to deliver localized radiation. Approximately 40,000 men in the United States receive this treatment each year.

The techniques for permanent interstitial prostate brachytherapy have evolved over time. Early techniques involved freehand placement of seeds in an open surgical procedure via the retropubic approach. Planning was based on the use of nomographs following intraoperative measurement of the size of the prostate gland.^2^, ^3^, ^4^ However, prostate brachytherapy has greatly improved with advances in imaging and treatment planning systems (TPSs). Current prostate brachytherapy implantation procedures are performed via the transperineal approach, using template‐guided and transrectal ultrasound (TRUS)‐guided needle insertion.^(5)^ The planning is performed using a three‐dimensional (3D) image‐based TPS. Although TRUS is commonly used for preoperative dosimetric planning, the use of computed tomography (CT) has also been noted in the literature. Post‐implant verification is typically done using fluoroscopy and CT.^6^, ^7^ Although CT is superior to TRUS and magnetic resonance imaging (MRI) for seed localization, it has limitations when delineating the boundaries of the prostate and the surrounding soft tissues.^8^, ^9^ MRI is well known for its ability to generate exquisite soft‐tissue contrast superior to that generated by CT or ultrasonography. Thus, if post‐implant prostate MR images are available, the MR images can be fused with CT images for post‐implant treatment planning and dosimetry.^(10)^

VariSeed 8.0, marketed by Varian Medical Systems, Inc. (Palo Alto, CA), is the prostate implant brachytherapy TPS used at our institution. VariSeed is commonly used for TRUS‐ and CT‐based treatment planning. Recently, interest in MRI‐based treatment planning has considerably increased owing to the ability of MRI to provide superior delineation of soft tissue. The American College of Radiology and the American Brachytherapy Society have particularly recommended MRI–CT fusion for post‐implant dosimetry. Currently, the VariSeed TPS has limited tools for this type of fusion registration. The MIM Symphony (MIM Software Inc., Cleveland, OH) LDR prostate brachytherapy TPS has recently been introduced into the market; this system provides better tools for MRI–TRUS fusion, MRI–CT fusion, or MRI‐only treatment planning. However, the dosimetric and treatment planning validation of this TPS is currently limited. The only other available study that compared VariSeed TPS with MIM Symphony was by Gossman et al.^(11)^ who evaluated dose‐volume histograms (DVHs) for I‐125 (Model 6711). Table 1 provides a brief comparison between these two TPSs.

In the present study, we sought to verify the MIM Symphony software more comprehensively by comparing its dosimetry and treatment planning accuracy with that of the formalism recommended by the American Association of Physicists in Medicine Task Group 43 (TG‐43), as well as with the universally used VariSeed TPS.

**Table 1:** Advantages and disadvantages of MIM Symphony TPS compared to VariSeed TPS

*MIM Symphony TPS (compared to VariSeed TPS)*	
Advantages	Multimodality treatment planning available based on MRI, CT, and US (compared to US and CT only capability in VariSeed)
	Enhanced MRI, CT, and US fusion capability
	Algorithm for autocontouring
	Plan library available for faster treatment planning
	Improved intraop dosimetry with needle shifts and deflections
	Dose summation with other plans (external beam and brachytherapy)
	Plans from multiple treatment planning system can be imported (VariSeed requires special VariSeed‐specific format)
Disadvantages	New TPS in the market
	Not as widely used in clinics

## II. MATERIALS AND METHODS

Two aspects of the MIM Symphony TPS system were evaluated. First, the line and point source models for each commissioned radioactive source were verified against independent dose calculations from the TG‐43 formalism and the clinical TPS currently used at our institution (VariSeed).^12^, ^13^ Second, the treatment planning‐related tests, such as the volume calculations of structures, text and plot printout, as well as the parameters from the DVHs used for assessing the goodness of the treatment plans, were evaluated to test the performance of the TPS and verify the planning process.

### A. Dosimetry validation

To check the accuracy of the MIM Symphony TPS dose calculations, we performed independent dose calculations using the TG‐43 formalism.^12^, ^13^ According to the TG‐43 formalism, the dose rate for brachytherapy sources using 2D source approximation can be obtained from the following equation:

(1) $$\dot{D}(r,\theta )=S_{k}\cdot \Lambda \cdot \frac{G_{L}(r,\theta )}{G_{L}(r_{0},\theta_{0})}\cdot g_{L}(r)\cdot F(r,\theta )$$

where $S_{k}$ is the air kerma strength, *Λ* is the dose‐rate constant, $G_{L}(r,\theta )$ is the geometry function, $G_{L}(r_{0},\theta_{0})$ is the geometry function at the reference point, $g_{L}(r)$ is the radial dose function, and F(r,θ) is the 2D anisotropy function. In the equation, *r* denotes the distance from the center of the active source to the point of interest; $r_{0}$ denotes the reference distance, which is specified to be 1 cm; and *θ* denotes the polar angle specifying the point of interest relative to the source longitudinal axis. The reference polar angle, $\theta_{0}$, defines the source transverse plane, and it is specified to be 90° or π/2 radians. It should be noted that subscript *L* is specified to imply line source approximation. For point source approximation, subscript *P* is used.

The constants are source‐specific and can be obtained from the TG‐43 models for certain source models or from the manufacturer. The radial function, $g_{L}(r)$, and the anisotropy function, F(r,θ), values are provided in the TG‐43 report for commonly used sources. The values for three out of the four LDR radioactive sources discussed here are consensus values from several sources obtained by measurements and verified by Monte Carlo simulations. The geometric function can be calculated as

(2) $$G_{L}(r,\theta )=\{\begin{array}{ll} \frac{\beta }{Lr\text{sin}\theta } & if\;\theta \neq 0^{\circ }\\ {\left(r^{2}-\frac{L^{2}}{4}\right)}^{-1} & if\;\theta =0^{\circ } \end{array}$$

where *L* is the active length of the source. Using Eq. (1) and half‐life ($T_{1/2}$) for an isotope, the dose at a point (r, θ) can be calculated as

(3) $$D(r,\theta )=\text{ln}(2)T_{1/2}\dot{D}(r,\theta )$$

MIM Symphony TPS and VariSeed TPS also provide users with an option to calculate doses using the point source approximation. To check the validity of the point source calculations in the TPS, we used 1D dose formalism from the TG‐43 report to perform independent dose calculations. 1D dose rate calculations are performed using this equation:

(4) $$\dot{D}(r)=S_{k}\cdot \Lambda \cdot \frac{G_{X}(r,\theta_{0})}{G_{X}(r_{0},\theta_{0})}\cdot g_{X}(r)\cdot \phi_{an}(r)$$

where, as mentioned above, the subscript *X* could be L for line source or P for point source, and $\phi_{an}(r)$ is the 1D anisotropy function. For point source approximation, $G_{P}(r)$ is equal to $r^{-2}$; therefore, the dose rate for point sources can be calculated as follows:

(5) $$\dot{D}(r)=S_{k}\cdot \Lambda \cdot {\left(\frac{r_{0}}{r}\right)}^{2}\cdot g_{P}(r)\cdot \phi_{an}(r)$$

**Figure 1:**
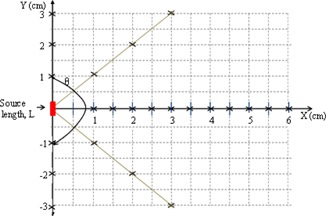
Schematic diagram of a two‐dimensional source with length L. The marked locations are points at which doses were calculated in the MIM Symphony treatment planning system and then compared with the calculations of the American Association of Physicists in Medicine (AAPM) Task Group (TG) 43 and the VariSeed treatment planning system calculations.

Doses for Cs‐131 (Proxcelan Model Cs‐1; IsoRay Medical Inc., Richmond, WA), Pd‐103 (Theraseed Model 200; Theragenics Corporation, Buford, GA), and two models of I‐125 sources (Oncoseed Model 6711; GE Healthcare, Arlington Heights, IL, and Advantage Model IAI‐125A; Isoaid, LLC, Port Richey, FL) used at our institution were calculated at various distances and angles using the 2D source approximation formalism, as shown in Fig. 1. All of these sources are available in the VariSeed TPS and MIM Symphony TPS for treatment planning purposes. The model numbers and characteristics of each of the sources are provided in Table 2. The values for constants, $g_{L}(r)$ and F(r,θ), were taken from the TG‐43 report for all models, except Cs‐131 for which currently no consensus data exists. For Cs‐131 the data was obtained from IsoRay Medical Inc. The MIM Symphony TPS also incorporates the same data. However, VariSeed uses slightly different parameters and are based on the results published by Rivard.^14^, ^15^

Doses based on point source approximation were also calculated for each source. The percentage dose differences were obtained between MIM Symphony TPS values and TG‐43 calculations, as well as between MIM Symphony TPS values and VariSeed TPS values.

**Table 2:** Model numbers and characteristics of each of the radioactive sources used for evaluation

	*Iodine‐125*	*Iodine‐125*	*Palladium‐103*	*Cesium‐131*
Model	IAI‐125A	6711	200	Cs‐1
Dose‐rate constant (Λ), cGy/h/U	0.981	0.965	0.686	1.059
Half‐life, days	59.399	59.399	16.991	9.689
Length, cm	0.45	0.456	0.45	0.45

### B. Treatment planning evaluation

As mentioned previously, VariSeed TPS is used at our institution for LDR prostate brachytherapy treatment planning. As part of our established clinical workflow, three treatment plans are generated for each patient during the course of the treatment. The pre‐implant plan is generated prior to treatment and is based on ultrasound images; this plan is used to determine the number of seeds, needles, and loading patterns. To determine the quality of the implantation, we perform a CT scan immediately following the implantation and use it to generate a second treatment plan, called the Day 0 treatment plan. After the prostate edema has resolved in approximately a month, another CT scan is performed to generate the Day 30 treatment plan.^16^, ^17^, ^18^, ^19^

For the pre‐implant plan, sonographic images of the prostate are acquired at 5 mm intervals using the Siemens Sonoline G20 ultrasound unit (Siemens Medical Solutions, Mountain View, CA), and the images are then transferred to the VariSeed TPS. The radiation oncologist contours the prostate, bladder, rectum, urethra, and seminal vesicles. A planning target volume (PTV) of 3 mm around the prostate volume is generated, except at the posterior aspect of the prostate along the rectum where no margin is added.

In order to check the volume accuracy of the contoured structured in MIM Symphony, a QA phantom (CIRS Brachytherapy QA Phantom Model 045 SN#D7210‐3; CIRS, Norfolk, VA) containing three ellipsoid objects with certified volumes was scanned. The images were imported into MIM Symphony and VariSeed and then contoured independently. The auto contouring tools of MIM Symphony TPS were used.

For the treatment planning study, we evaluated 25 pre‐implant plans and 25 Day 30 plans, using both planning systems for each of the two commonly used source models at our institution (I‐125 (Model 6711) and Pd‐103 (Model 200)). Comparisons were made for the volumes and DVH parameters of the prostate, bladder, and rectum. Note that, unlike the prostate, the bladder and rectum are partially contoured because these anatomical structures are not fully acquired on the ultrasound imaging system. The DVH parameters examined included D90, V100, V150, and V200 for the prostate and V50, V100, and V150 for the bladder and rectum. D90 is the minimum dose to 90% of the prostate and Vx is the volume receiving at least x% of the prescribed dose.

Because the plans in the VariSeed TPS were the actual clinical plans used for treatment, the DVH and volume values from this TPS were directly taken from the clinical plans. For the MIM Symphony‐based planning, the seed locations and contours were exported from the VariSeed TPS to the MIM Symphony TPS. From these 2D contours and seed coordinates, the volume and dose calculations were obtained. The line model was specified for dose calculations in both TPSs. In VariSeed, the dose matrix was specified with an XY resolution of 1 mm and a Z resolution to match the distance between the images (5 mm for pre‐implant plans and 2.5 mm for Day 30 plans). In MIM Symphony, the XY resolution for dose calculations was determined from the resolution of the contours exported from VariSeed. Although the XY resolution for the version of MIM Symphony we used was matched to the VariSeed resolution, the same was not true of the Z resolution for Day 30 plans. The version of MIM Symphony we evaluated provided only three options for dose calculation resolution in Z: 1 mm, 5/3 mm, and 5 mm. For resolution in the Z direction we used 5 mm for pre‐implant plans, while 5/3 mm was used for Day 30 plans since this was closest to the 2.5 mm resolution used for Day 30 plans in VariSeed.

For each DVH parameter evaluated, the difference was calculated between the values from MIM Symphony and VariSeed. A positive difference indicated that MIM Symphony calculated a greater value than VariSeed, and a negative difference indicated that VariSeed calculated a greater value than MIM Symphony.

## III. RESULTS

### A. Dosimetry validation

3, 4, 5, 6, 7, 8, 9, 10 show 2D and 1D dose calculations for Cs‐131 (Model Cs‐1), Pd‐103 (Model 200), I‐125 (Model 6711), and I‐125 (Model IAI‐125A). The values for constants, $g_{L}(r)$ and F(r,θ), were taken from the TG‐43 report for all models, except Cs‐131 for which the Cs‐131 source data sheet from the manufacturer was used. Dose calculations were performed for several different planes at various radial distances. The MIM Symphony TPS results were within 0.5% (or 0.02 cGy) for r>1 cm using the 2D formalism for most of the points, except for low‐dose gradient regions where even small differences in dose yielded a large percentage difference. The MIM Symphony values were also compared with VariSeed values, and these results were also within 0.5% (0.09 Gy) for r>1 cm. Similarly, for the 1D formalism, the MIM Symphony values agreed within 1.4% and 1% to TG‐43 and VariSeed, respectively, for r>1 cm.

**Table 3:** Dose calculations comparison between MIM Symphony, TG‐43, and VariSeed for cesium‐131 (Model‐Cs1) using line approximation model

							*% Dose Difference*	
*θ (deg)*	*X (cm)*	*Y (cm)*	*r (cm)*	*Dose (cGy)*			*MIM vs. TG‐43*	*MIM vs. VariSeed*
				*MIM*	*TG‐43*	*VariSeed*		
90	1.00	0.00	1.00	353.26	353.26	355.01	0.00	−0.49
90	1.50	0.00	1.50	152.13	152.13	152.89	0.00	−0.50
90	2.00	0.00	2.00	80.98	80.98	81.38	0.00	−0.49
90	2.50	0.00	2.50	48.27	48.26	48.38	0.02	−0.23
90	3.00	0.00	3.00	30.85	30.85	31.01	−0.02	−0.52
90	3.50	0.00	3.50	20.69	20.69	20.81	−0.02	−0.58
90	4.00	0.00	4.00	14.35	14.35	14.42	0.00	−0.49
90	4.50	0.00	4.50	10.21	10.21	10.30	−0.03	−0.87
90	5.00	0.00	5.00	7.41	7.41	7.45	−0.03	−0.54
90	5.50	0.00	5.50	5.47	5.47	5.52	0.06	−0.91
90	6.00	0.00	6.00	4.08	4.08	4.10	−0.12	−0.49
0	0.00	1.00	1.00	310.57	310.57	312.11	0.00	−0.49
0	0.00	2.00	2.00	68.86	68.86	69.20	0.01	−0.49
0	0.00	3.00	3.00	26.13	26.13	26.26	−0.02	−0.50
45	1.00	1.00	1.41	165.23	165.25	166.10	−0.01	−0.52
45	2.00	2.00	2.83	34.13	34.11	34.29	0.07	−0.47
45	3.00	3.00	4.24	11.59	11.60	11.69	−0.11	−0.86
135	1.00	−1.00	1.41	165.23	165.25	166.10	−0.01	−0.52
135	2.00	−2.00	2.83	34.13	34.11	34.29	0.07	−0.47
135	3.00	−3.00	4.24	11.59	11.60	11.69	−0.11	−0.86
180	0.00	−1.00	1.00	310.57	310.57	312.11	0.00	−0.49
180	0.00	−2.00	2.00	68.86	68.86	69.20	0.01	−0.49
180	0.00	−3.00	3.00	26.13	26.13	26.26	−0.02	−0.50

**Table 4:** Dose calculations comparison between MIM Symphony, TG‐43, and VariSeed for cesium‐131 (Model‐Cs1) using point approximation model

							*% Dose Difference*	
*θ (deg)*	*X (cm)*	*Y (cm)*	*r (cm)*	*Dose (cGy)*			*MIM vs. TG‐43*	*MIM vs. VariSeed*
				*MIM*	*TG‐43*	*VariSeed*		
90	1.00	0.00	1.00	341.25	341.25	342.94	0.00	−0.49
90	1.50	0.00	1.50	146.20	145.15	146.93	0.73	−0.50
90	2.00	0.00	2.00	77.74	76.98	78.13	0.98	−0.50
90	2.50	0.00	2.50	46.37	45.84	46.46	1.16	−0.19
90	3.00	0.00	3.00	29.65	29.31	29.80	1.16	−0.50
90	3.50	0.00	3.50	19.90	19.67	20.02	1.15	−0.60
90	4.00	0.00	4.00	13.82	13.65	13.89	1.25	−0.50
90	4.50	0.00	4.50	9.83	9.71	9.92	1.20	−0.91
90	5.00	0.00	5.00	7.14	7.05	7.18	1.30	−0.56
90	5.50	0.00	5.50	5.26	5.20	5.32	1.18	−1.13
90	6.00	0.00	6.00	3.94	3.89	3.95	1.40	−0.25

**Table 5:** Dose calculations comparison between MIM Symphony, TG‐43, and VariSeed for palladium‐103 (Model‐200) using line approximation model

							*% Dose Difference*	
*θ (deg)*	*X (cm)*	*Y (cm)*	*r (cm)*	*Dose (cGy)*			*MIM vs. TG‐43*	*MIM vs. VariSeed*
				*MIM*	*TG‐43*	*VariSeed*		
90	1.00	0.00	1.00	403.58	403.58	402.83	0.00	0.19
90	1.50	0.00	1.50	135.43	135.43	135.18	0.00	0.18
90	2.00	0.00	2.00	56.61	56.61	56.51	0.00	0.18
90	2.50	0.00	2.50	26.80	26.80	26.75	0.00	0.19
90	3.00	0.00	3.00	13.72	13.72	13.69	0.01	0.22
90	3.50	0.00	3.50	7.45	7.45	7.43	0.05	0.27
90	4.00	0.00	4.00	4.17	4.17	4.16	0.04	0.24
90	4.50	0.00	4.50	2.42	2.44	2.54	−0.72	−4.72
90	5.00	0.00	5.00	1.45	1.45	1.45	−0.15	0.00
90	5.50	0.00	5.50	0.89	0.89	0.92	0.48	−3.26
90	6.00	0.00	6.00	0.55	0.55	0.55	0.35	0.00
90	6.50	0.00	6.50	0.34	0.34	0.36	0.13	−5.56
90	7.00	0.00	7.00	0.22	0.22	0.22	0.50	0.00
0	0.00	1.00	1.00	231.93	231.93	231.50	0.00	0.19
0	0.00	2.00	2.00	30.23	30.23	30.17	0.01	0.20
0	0.00	3.00	3.00	6.96	6.96	6.95	−0.01	0.14
45	1.00	1.00	1.41	128.17	128.52	128.70	−0.28	−0.41
45	2.00	2.00	2.83	14.01	14.00	14.14	0.07	−0.92
45	3.00	3.00	4.24	2.65	2.68	2.74	−0.97	−3.28
135	1.00	−1.00	1.41	128.17	128.52	128.70	−0.28	−0.41
135	2.00	−2.00	2.83	14.01	14.00	14.14	0.07	−0.92
135	3.00	−3.00	4.24	2.65	2.68	2.74	−0.97	−3.28
180	0.00	−1.00	1.00	231.93	231.93	231.50	0.00	0.19
180	0.00	−2.00	2.00	30.23	30.23	30.17	0.01	0.20
180	0.00	−3.00	3.00	6.96	6.96	6.95	−0.01	0.14

**Table 6:** Dose calculations comparison between MIM Symphony, TG‐43, and VariSeed for palladium‐103 (Model‐200) using point approximation model

							*% Dose Difference*	
*θ (deg)*	*X (cm)*	*Y (cm)*	*r (cm)*	*Dose (cGy)*			*MIM vs. TG‐43*	*MIM vs. VariSeed*
				*MIM*	*TG‐43*	*VariSeed*		
90	1.00	0.00	1.00	345.06	345.06	344.42	0.00	0.19
90	1.50	0.00	1.50	116.81	116.56	116.58	0.22	0.20
90	2.00	0.00	2.00	49.25	49.24	49.15	0.01	0.20
90	2.50	0.00	2.50	23.50	23.51	23.46	−0.04	0.17
90	3.00	0.00	3.00	12.13	12.13	12.11	0.00	0.17
90	3.50	0.00	3.50	6.62	6.63	6.61	−0.17	0.15
90	4.00	0.00	4.00	3.73	3.72	3.72	0.14	0.27
90	4.50	0.00	4.50	2.17	2.18	2.27	−0.57	−4.41
90	5.00	0.00	5.00	1.30	1.30	1.30	−0.25	0.00
90	5.50	0.00	5.50	0.80	0.80	0.83	0.40	−3.61
90	6.00	0.00	6.00	0.50	0.49	0.50	1.08	0.00

**Table 7:** Dose calculations comparison between MIM Symphony, TG‐43, and VariSeed for iodine‐125 (Model 6711) using line approximation model

							*% Dose Difference*	
*θ (deg)*	*X (cm)*	*Y (cm)*	*r (cm)*	*Dose (cGy)*			*MIM vs. TG‐43*	*MIM vs. VariSeed*
				*MIM*	*TG‐43*	*VariSeed*		
90	1.5	0	1.50	804.23	804.23	802.74	0.00	0.19
90	2	0	2.00	406.13	406.13	405.38	0.00	0.19
90	2.5	0	2.50	229.72	229.84	230.6	−0.05	−0.38
90	3	0	3.00	140.29	140.29	140.03	0.00	0.19
90	3.5	0	3.50	91.48	91.56	91.83	−0.09	−0.38
90	4	0	4.00	61.95	61.95	61.84	−0.01	0.18
90	4.5	0	4.50	42.25	42.14	42.36	0.25	−0.26
90	5	0	5.00	29.1	29.10	29.05	−0.01	0.17
90	5.5	0	5.50	20.64	20.71	20.91	−0.33	−1.29
90	6	0	6.00	14.99	14.99	14.97	−0.02	0.13
90	6.5	0	6.50	10.98	10.97	11.08	0.11	−0.90
90	7	0	7.00	8.12	8.12	8.1	0.01	0.25
0	0	1	1.00	756.84	756.84	755.44	0.00	0.19
0	0	2	2.00	180.87	180.87	180.53	0.00	0.19
0	0	3	3.00	68.69	68.69	68.56	0.00	0.19
45	1	1	1.41	879.29	880.03	877.43	−0.08	0.21
45	2	2	2.83	156.5	156.40	156.93	0.06	−0.27
45	3	3	4.24	48.83	48.81	48.81	0.04	0.04
135	1	−1	1.41	879.29	880.03	877.43	−0.08	0.21
45	2	2	2.83	156.5	156.40	156.93	0.06	−0.27
135	3	−3	4.24	48.83	48.81	48.81	0.04	0.04
180	0	−1	1.00	756.84	756.84	755.44	0.00	0.19
180	0	−2	2.00	180.87	180.87	180.53	0.00	0.19
180	0	−3	3.00	68.69	68.69	68.56	0.00	0.19

**Table 8:** Dose calculations comparison between MIM Symphony, TG‐43, and VariSeed for iodine‐125 (Model 6711) using point approximation model

							*% Dose Difference*	
*θ (deg)*	*X (cm)*	*Y (cm)*	*r (cm)*	*Dose (cGy)*			*MIM vs. TG‐43*	*MIM vs. VariSeed*
				*MIM*	*TG‐43*	*VariSeed*		
90	1.5	0	1.50	757.99	757.57	756.80	0.06	0.16
90	2	0	2.00	382.17	382.39	381.68	−0.06	0.13
90	2.5	0	2.50	216.28	216.43	217.10	−0.07	−0.38
90	3	0	3.00	132.15	132.12	131.87	0.03	0.21
90	3.5	0	3.50	86.22	86.23	86.50	−0.01	−0.32
90	4	0	4.00	58.42	58.37	58.26	0.09	0.27
90	4.5	0	4.50	39.86	39.76	39.97	0.24	−0.28
90	5	0	5.00	27.47	27.50	27.45	−0.12	0.07

**Table 9:** Dose calculations comparison between MIM Symphony, TG‐43, and VariSeed for iodine‐125 (Model IAI‐125A) using line approximation model

							*% Dose Difference*	
*θ (deg)*	*X (cm)*	*Y (cm)*	*r (cm)*	*Dose (cGy)*			*MIM vs. TG‐43*	*MIM vs. VariSeed*
				*MIM*	*TG‐43*	*VariSeed*		
90	1.50	0.00	1.50	812.16	812.16	810.66	0.00	0.19
90	2.00	0.00	2.00	405.77	405.77	405.02	0.00	0.19
90	2.50	0.00	2.50	228.00	227.78	228.75	0.10	−0.33
90	3.00	0.00	3.00	137.88	137.88	137.62	0.00	0.19
90	3.50	0.00	3.50	88.41	88.53	89.30	−0.13	−1.00
90	4.00	0.00	4.00	59.43	59.43	59.32	0.01	0.19
90	4.50	0.00	4.50	41.51	41.53	41.86	−0.05	−0.84
90	5.00	0.00	5.00	29.91	29.91	29.86	0.00	0.17
90	5.50	0.00	5.50	22.10	22.11	22.19	−0.06	−0.41
90	6.00	0.00	6.00	16.60	16.60	16.57	0.02	0.18
0	0.00	1.00	1.00	844.25	844.25	842.68	0.00	0.19
0	0.00	2.00	2.00	201.55	201.55	201.18	0.00	0.18
0	0.00	3.00	3.00	71.94	71.94	71.80	0.00	0.19
45	1.00	1.00	1.41	899.42	900.99	895.96	−0.17	0.39
45	2.00	2.00	2.83	153.38	153.24	153.69	0.09	−0.20
45	3.00	3.00	4.24	47.05	46.91	47.32	0.30	−0.57
135	1.00	−1.00	1.41	899.42	900.99	895.96	−0.17	0.39
135	2.00	−2.00	2.83	153.38	153.24	153.69	0.09	−0.20
135	3.00	−3.00	4.24	47.05	46.91	47.32	0.30	−0.57
180	0.00	−1.00	1.00	844.25	844.25	842.68	0.00	0.19
180	0.00	−2.00	2.00	201.55	201.55	201.18	0.00	0.18
180	0.00	−3.00	3.00	71.94	71.94	71.80	0.00	0.19

**Table 10:** Dose calculations comparison between MIM Symphony, TG‐43, and VariSeed for iodine‐125 (Model IAI‐125A) using point approximation model

							*% Dose Difference*	
*θ (deg)*	*X (cm)*	*Y (cm)*	*r (cm)*	*Dose (cGy)*			*MIM vs. TG‐43*	*MIM vs. VariSeed*
				*MIM*	*TG‐43*	*VariSeed*		
90	1.50	0.00	1.50	784.55	785.36	783.34	−0.10	0.15
90	2.00	0.00	2.00	391.16	390.94	390.21	0.06	0.24
90	2.50	0.00	2.50	218.77	218.30	219.35	0.21	−0.26
90	3.00	0.00	3.00	131.67	131.66	131.42	0.00	0.19
90	3.50	0.00	3.50	84.52	84.57	85.34	−0.06	−0.96
90	4.50	0.00	4.50	39.76	39.81	40.11	−0.14	−0.87
90	5.00	0.00	5.00	28.69	28.71	28.66	−0.08	0.10
90	5.50	0.00	5.50	21.17	21.21	21.27	−0.17	−0.47
90	6.00	0.00	6.00	15.88	15.89	15.85	−0.08	0.19

### B. Treatment planning validation

The CIRS Brachytherapy QA phantom results yielded less than 1% difference between the volume calculated by MIM Symphony and actual volume of the phantom. Comparisons were also made to the volumes calculated by VariSeed. Table 11 summarizes the results for different structures.

**Figure 2:**
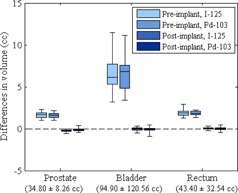
Differences between the volumes calculated in MIM Symphony and those calculated in VariSeed for the prostate, bladder, and rectum. Dose‐volume histogram data for iodine‐125 (I‐125; Model 6711) and palladium‐103 (Pd‐103; Model 200) are shown. The bottom and top of each box are the first and third quartiles, the line inside the box indicates the median, and the ends of the whiskers indicate the minimum and maximum values. Total volumes are shown as mean ±SD.

**Figure 3:**
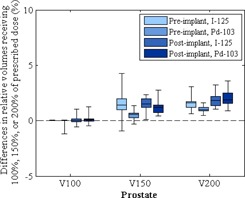
Differences between the prostate V100, V150, and V200 calculations in MIM Symphony and those in VariSeed. Vx= portion of the total prostate volume receiving ≥x% of the prescribed dose. Dose‐volume histogram data for iodine‐125 (I‐125; Model 6711) and palladium‐103 (Pd‐103; Model 200) are shown. The bottom and top of each box are the first and third quartiles, the line inside the box indicates the median, and the ends of the whiskers indicate the minimum and maximum values.

Figure 2 shows the differences between the volumes calculated in MIM Symphony and those calculated in VariSeed for clinical cases. Figure 3 shows the differences between the prostate V100, V150, and V200 values calculated in MIM Symphony and those calculated in VariSeed. Because the prostate V100, V150, and V200 values were recorded as percentages of the total prostate volume, the differences in these values are reported in percentages as well. Mean, standard deviation (SD), and minimum and maximum differences in prostate D90 values are shown in Table 12

**Table 11:** Volume calculations of known structures by MIM Symphony and VariSeed

				*% Volume Difference*	
		*Volumes (cc)*		*MIM*	
	*Actual*	*MIM Symphony*	*VariSeed*	*Symphony vs. Actual*	*VariSeed vs. Actual*
Small	3.45	3.43	3.47	−0.6	0.6
Medium	8.51	8.57	8.45	0.7	−0.7
Large	19.78	19.89	20.06	0.6	1.4

**Figure 4:**
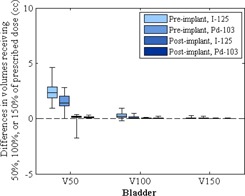
Differences between the bladder V50, V100, and V150 calculations in MIM Symphony and those in VariSeed. Vx= portion of the bladder volume receiving ≥x% of the prescribed dose. Dose‐volume histogram data for iodine‐125 (I‐125; Model 6711) and palladium‐103 (Pd‐103; Model 200) are shown. The bottom and top of each box are the first and third quartiles, the line inside the box indicates the median, and the ends of the whiskers indicate the minimum and maximum values.

**Figure 5:**
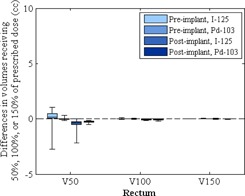
Differences between the rectum V50, V100, and V150 calculations in MIM Symphony and those in VariSeed. Vx= portion of the rectal volume receiving ≥x% of the prescribed dose. Dose‐volume histogram data for iodine‐125 (I‐125; Model 6711) and palladium‐103 (Pd‐103; Model 200) are shown. The bottom and top of each box are the first and third quartiles, the line inside the box indicates the median, and the ends of the whiskers indicate the minimum and maximum values.

4, 5 show the differences in the V50, V100, and V150 values calculated for the bladder and rectum (reported in cubic centimeters). The DVH parameters calculated by the two TPSs did not differ substantially. Mean differences in prostate V100, V150, and V200 values were within 2% (Fig. 3); mean differences in bladder V50, V100, and V150 values were within 2 cc (Fig. 4); mean differences in rectum V50, V100 and V150 values were within 0.5 cc (Fig. 5).

**Table 12:** Differences between the prostate D90 calculations in MIM Symphony and those in VariSeed

	*Differences in Prostate D90 (Gy)*			
	*Pre‐implant*		*Day 30*	
	*I‐125*	*Pd‐103*	*I‐125*	*Pd‐103*
Mean	0.73	0.18	1.46	1.99
SD	0.64	0.68	1.40	1.29
Minimum	−1.35	−2.52	−0.32	−0.23
Maximum	2.70	0.85	6.80	5.75

The prescribed dose for iodine‐125 (I‐125) implants was 145 Gy, and for palladium‐103 (Pd‐103), 125 Gy. D90= minimum dose received by 90% of the prostate volume.

## IV. DISCUSSION

In this report, we evaluated the MIM symphony TPS for LDR prostate brachytherapy treatment planning and compared its performance, data, and results with the VariSeed TPS. Table 13 provides a detailed overview of the tests performed based on the recommendations presented in TG‐53 report.^(20)^

In our analysis, a comprehensive set of dose calculations for all commissioned isotopes and models at our institution in the MIM Symphony TPS were in agreement with those obtained from the VariSeed TPS, as well as with those calculated using the formalism recommended by TG‐43. The results were within 0.5% (0.09 Gy) for most points for r>1 cm using both the 1D and 2D formalism. The dose calculation accuracy for the MIM Symphony TPS was limited for r<1cm; however, a similar discrepancy was observed with VariSeed for r<1cm when compared to the TG‐43 calculations.

Our results showed that the volume calculations for the prostate and rectum did not differ substantially. The bladder showed higher volume differences between MIM Symphony and VariSeed than the other two structures in the pre‐implant plans. We believe these differences are due to the large Z direction resolution (5 mm used in both MIM Symphony and VariSeed), large contoured areas, partial contouring (bladder contours on a few slices only), and interpolation of VariSeed data. Furthermore, the differences in bladder V50 values are slightly higher. The greater volume differences calculated on pre‐implant plans compared with Day 30 plans are attributable to XY‐plane resolution differences between the pre‐implant ultrasound images and the finer‐resolution Day 30 CT images. On the other hand, as noted above, the Z resolution for the Day 30 plans in MIM Symphony was 5/3 mm compared with 2.5 mm in VariSeed. We believe this discrepancy in resolution also caused differences in Day 30 comparisons between the two systems.

Overall, the differences in the planning parameters evaluated in this work do not disqualify MIM Symphony version 5.4 from clinical treatment planning, but these differences do caution users to carefully evaluate the contours when importing plans from VariSeed. These issues will not be present if the entire treatment planning is done with the MIM Symphony software. Therefore, we believe the MIM Symphony TPS has the potential to play a role in the clinical treatment planning of LDR prostate brachytherapy.

**Table 13:** Evaluation of the MIM Symphony TPS based on the guidelines presented in the TG‐53 report.^(20)^

Dose calculation accuracy with respect to TG‐43 and another commercial treatment planning system
Source input and geometrical accuracy
Source display
Optimization and evaluation
Verify properties or attributes for each source in the library
Benchmark tests which confirm the entire process used for brachytherapy planning for each basic kind of brachytherapy procedure
Text printout (e.g., treatment machine/modality/source/energy, beam parameters, calculation algorithm, grid size, dose to and position of calculation points, software version)
2D dose plots (e.g., location/orientation of displayed plane, patient contour, dose information, scale factor, and location of calculation points)
DVHs (plot legend, scales and units, anatomical structures, case, plan, and other identifying info)
Data transfer between different TPSs
Accuracy of volume calculations
Template grid accuracy

## V. CONCLUSIONS

In the present study, we compared two prostate brachytherapy TPSs: Varian VariSeed 8.0 and MIM Symphony 5.4. We found no substantial differences in the dosimetry calculations between the two TPSs; the volume and DVH calculations for the prostate, rectum, and bladder did not differ. On the basis of these results, we conclude that MIM Symphony TPS can be used as an alternative to VariSeed TPS for LDR prostate brachytherapy.

## ACKNOWLEDGMENTS

The authors would like to thank Erica Goodoff from the Department of Scientific Publications at the University of Texas MD Anderson Cancer Center for providing editorial assistance.

## Supporting information

Supplementary Material

## References

[1] American Cancer Society. Cancer facts & figures 2013. Atlanta, GA: American Cancer Society; 2013.

[2] Anderson LL . Spacing nomograph for interstitial implants of 125 I seeds. Med Phys. 1976;3(1):48–51. 1244190 10.1118/1.594269

[3] Anderson LL , Moni JV , Harrison LB . A nomograph for permanent implants of palladium‐103 seeds. Int J Radiat Oncol Biol Phys. 1993;27(1):129–35. 8365933 10.1016/0360-3016(93)90430-4

[4] Henschke UK and Ceve P . Dimension averaging a simple method for dosimetry of interstitial implants. Radiobiol Radiother. 1968;9(3):287–98. 5683780

[5] Yu Y , Anderson LL , Li Z , Mellenberg DE , et al. Permanent prostate seed implant brachytherapy: report of the American Association of Physicists in Medicine Task Group Report No. 64. Med Phys. 1999;26(76):2054–76. 10535622 10.1118/1.598721

[6] Wallner K , Chiu‐Tsao ST , Roy J , et al. An improved method for computerized tomography‐planned transperineal 125‐iodine prostate implants. J Urol. 1991;146(1):90–95. 1711591 10.1016/s0022-5347(17)37721-2

[7] Wallner K , Roy J , Zelefsky M , Fuks Z , Harrison L . Fluoroscopic visualization of the prostatic urethra to guide transperineal prostate implantation. Int J Radiat Oncol Biol Phys. 1994;29(4):863–67. 8040035 10.1016/0360-3016(94)90577-0

[8] Albert J , Swanson D , Pugh TJ , et al. Magnetic resonance imaging‐based treatment planning for prostate brachytherapy. Brachytherapy. 2013;12(1):30–37. 22727474 10.1016/j.brachy.2012.03.009

[9] Frank SJ , Stafford RJ , Bankson JA , et al. A novel MRI marker for prostate brachytherapy. Int J Radiat Oncol Biol Phys. 2008;71(1):5–8. 18406882 10.1016/j.ijrobp.2008.01.028

[10] Brown AP , Pugh TJ , Swanson DA , et al. Improving prostate brachytherapy quality assurance with MRI‐CT fusion‐based sector analysis in a phase II prospective trial of men with intermediate‐risk prostate cancer. Brachytherapy. 2013;12(5):401–07. 23380383 10.1016/j.brachy.2012.10.001

[11] Gossman MS , Hancock SS , Kudchadker RJ , Lundahl PR , Cao M , Melhus CS . Brachytherapy dose‐volume histogram commissioning with multiple planning systems. J Appl Clin Med Phys. 2014;15(2):110–20. 10.1120/jacmp.v15i2.4620 PMC5875493 24710449

[12] Nath R , Anderson LL , Luxton GK , Weaver KA , Williamson JF , Meigooni AS . Dosimetry of interstitial brachytherapy sources: recommendations of the AAPM Radiation Therapy Committee Task Group No. 43. Med Phys. 1995;22(2):209–34. 7565352 10.1118/1.597458

[13] Rivard MJ , Coursey BM , DeWerd LA , et al. Update of AAPM Task Group No. 43 report: a revised AAPM protocol for brachytherapy dose calculations. Med Phys. 2004;31(3): 633–74. 15070264 10.1118/1.1646040

[14] Rivard MJ . Brachytherapy dosimetry parameters calculated for a 131Cs source. Med Phys. 2007;34(2):754–62. 17388193 10.1118/1.2432162

[15] Rivard MJ . Erratum: Brachytherapy dosimetry parameters calculated for a 131Cs source. Med Phys. 2009;36(1):279. 10.1118/1.2432162 17388193

[16] Kovtun KA , Wolfsberger L , Niedermayr T , et al. Dosimetric quality and evolution of edema after low‐dose‐rate brachytherapy for small prostates: implications for the use of newer isotopes. Brachytherapy. 2014;13(2):152–56. 23911279 10.1016/j.brachy.2013.05.006

[17] Tejwani A , Bieniek E , Puckett L , et al. Case series analysis of post‐brachytherapy prostate edema and its relevance to post‐implant dosimetry. Post‐implant prostate edema and dosimetry. J Contemp Brachytherapy. 2012;4(2):75–80. 23349648 10.5114/jcb.2012.29363 PMC3552628

[18] Slobada RS , Usmani N , Monajemi TT , Liu DM . Impact of edema and seed movement on the dosimetry of prostate seed implants. J Med Phys. 2012;37(2):81–89. 22557797 10.4103/0971-6203.94742 PMC3339147

[19] Slobada RS , Usmani N , Pedersen J , Murtha A , Pervez N , Yee D . Time course of prostatic edema post permanent seed implant determined by magnetic resonance imaging. Brachytherapy. 2010;9(4):354–61. 20116344 10.1016/j.brachy.2009.09.008

[20] Fraass IB , K Doppke , M Hunt , et al., American Association of Physicists in Medicine Radiation Therapy Committee Task Group 53: Quality assurance for clinical radiotherapy treatment planning. Med Phys. 1998;25(10):1773–829. 9800687 10.1118/1.598373

