# Supplementary material for: Evaluation of the MIM Symphony treatment planning system for low‐dose‐rate‐ prostate brachytherapy
Source: J Appl Clin Med Phys. 2015 Sep 8;16(5):62–75. doi: 10.1120/jacmp.v16i5.5057 (PMC5690179; doi:10.1120/jacmp.v16i5.5057)
Supplement: Supplementary file 1 — Supplementary Material [file ACM2-16-062-s001.docx]

**Commissioning and verification of the MIM Symphony treatment planning system for low dose rate prostate brachytherapy**

Sandeep Dhanesar, PhD^1^, Tze Yee Lim, BS,^2^ Weiliang Du,^1^ PhD, Teresa L. Bruno,^1^ BS, CMD Steven J. Frank, MD^3^ and Rajat J. Kudchadker, PhD^1^

*^1^Department of Radiation Physics, The University of Texas MD Anderson Cancer Center, 1515 Holcombe Boulevard, Unit 94, Houston, TX 77030, USA*

*^2^The University of Texas at Houston Graduate School of Biomedical Sciences, 6767 Bertner Avenue, S3.8344, Houston, TX 77030, USA*

*^3^Department of Radiation Oncology, The University of Texas MD Anderson Cancer Center, 1515 Holcombe Boulevard, Houston, TX 77030, USA*

Corresponding Author: Rajat J. Kudchadker, Department of Radiation Physics, Unit 1202, The University of Texas MD Anderson Cancer Center, 1515 Holcombe Boulevard, Houston, TX 77030, USA; Phone: 713-563-2538; Fax: 713-563-6949

rkudchad@mdanderson.org

Short Title: MIM Symphony treatment planning for prostate brachytherapy.
